# Using an Online Vaccination Registry to Confirm Tetanus Status in Children with Tetanus-prone Wounds

**DOI:** 10.5811/westjem.2020.6.46582

**Published:** 2020-08-21

**Authors:** Cristina Zeretzke-Bien, Janelle McCall, Todd Wylie, Muhammad A.B. Chowdhury, Meenakshi Balakrishnan, Phyllis Hendry, Colleen J. Kalynych, Hac-Tu J. Chung

**Affiliations:** * University of Florida in Gainesville, Department of Emergency Medicine, Gainesville, Florida; † University of Texas Southwestern, Department of Emergency Medicine, Dallas, Texas; ‡ University of Florida in Jacksonville, Department of Emergency Medicine, Jacksonville, Florida; § University of Florida in Jacksonville, Office of Educational Affairs, Jacksonville, Florida

## Abstract

**Introduction:**

Tetanus vaccination status is an important consideration for emergency physicians managing patients with tetanus-prone wounds. Physicians must identify at-risk patients, but vaccination histories are often unknown and commonly lack documentation. The study objective was to determine the potential impact of an online immunization registry (Florida SHOTS – State Health Online Tracking System) on the appropriate administration of tetanus prophylaxis for pediatric patients managed in the emergency department (ED).

**Methods:**

We conducted a retrospective review of all patients less than 18 years old who received ED tetanus prophylaxis at two separate sites between January 2011–May 2015. The Florida SHOTS database was accessed to determine vaccination status for each patient in the study group at the time of the encounter. We compared vaccination status for each patient, as documented in the electronic health record (EHR), with Florida SHOTS data to determine whether tetanus prophylaxis was indicated. The proportion of patients receiving tetanus prophylaxis in the ED, who were subsequently identified as up to date with tetanus vaccination per Florida SHOTS, was determined.

**Results:**

We identified 743 patients who received ED tetanus prophylaxis. Forty-three (6%) were listed as “up to date” on the EHR and 656 (93%) were listed as “not up to date.” In comparison, 209 (30%) of the study group were identified as “up to date” via Florida SHOTS, and 477 (70%) were not. We accessed the Florida SHOTS record retrospectively to determine whether the vaccine was required. It was determined that 174 (25%) of the patients received tetanus prophylaxis unnecessarily as they were already up to date per Florida SHOTS documentation.

**Conclusion:**

Twenty-five percent of patients vaccinated for tetanus in the ED could have been spared if Florida SHOTS data had been used by providers at the time of the encounter. Access to Florida SHOTS provides valuable information regarding vaccination status that impacts patient care and resource utilization in the ED.

## INTRODUCTION

Tetanus is a life-threatening disease caused by the bacterium *Clostridium tetani*. Mortality is high in those who are not immunized and do not receive treatment. Thankfully, it is now rare in the developed world due to tetanus vaccination programs. Widespread use of tetanus-toxoid containing vaccines and tetanus immune globulin (TIG) for wound management has led to a 95% decline in the number of tetanus cases and a 99% decrease in the number of tetanus-related deaths since the 1940s.^1^ However, to achieve and maintain appropriate immunization status, children must receive the complete primary series of tetanus vaccinations and subsequent booster vaccinations as indicated. Currently, the US Centers for Disease Control and Prevention (CDC) recommend that the DTaP (diphtheria, tetanus, and acellular pertussis vaccine) be given as a 5-dose series at ages 2, 4, and 6 months, as well as at ages 15–18 months and 4–6 years. Tdap (tetanus, diphtheria, and pertussis) is administered at 11–12 years of age. Following that, a Td booster should be given every 10 years.

In the emergency department (ED), it is recommended to give the tetanus vaccine under the following conditions: if tetanus vaccine status is unknown; if the patient has had less than three doses (would also receive TIG for dirty wound), or if the patient has had at least three doses but it has been over five years since the last dose (10 years for “clean” wound; but of note, most wounds are considered “dirty wounds” in the ED).^1^ Tetanus vaccination status is an important consideration for emergency physicians (EP) managing pediatric patients with tetanus-prone wounds. EPs must decide which patients are at risk for tetanus based on their vaccination status; however, vaccination histories are often unknown by parents and/or caretakers and commonly lack documentation.^2^ A recent study suggests that multiple formulations of tetanus vaccinations and fragmented documentation of immunizations increase the prevalence of medication errors related to tetanus vaccinations.^3^

Most of the literature surrounding ED tetanus vaccination demonstrates inaccuracies of judgment of patients’ tetanus vaccination status. A recent pediatric study in Utah showed that providers incorrectly assessed tetanus vaccination status 8.8% of the time.^4^ Of these, 85% (7.4% of the entire group) were incorrectly identified as being up to date. Therefore, if they had a clinical indication for the tetanus vaccine, providers would have missed giving it. A longitudinal study in Taiwan following 770,000 adult patients over eight years discovered that more than 160,000 unnecessary tetanus boosters were given.^5^

In an effort to provide all practitioners with access to up-to-date vaccination records, the CDC has supported initiatives to develop local and state immunization registries. The CDC reports that every state in the union is either developing or operating a state or regional immunization registry. Florida SHOTS (State Health Online Tracking System) is a free, statewide, centralized online vaccination registry, which was created in 2003. The registry is an online Health Insurance Portability and Accountability Act (HIPAA)-exempt immunization registry available to all healthcare providers (Florida Statute, Section 456.057). Once an account is set up, the database is easily accessible to medical personnel in the ED as well as in outpatient clinics. This is a centralized database tracking vaccine administration, which can be accessed easily by healthcare providers, as opposed to individual hospital or clinic EHRs, which may or may not communicate with each other.

Presumably, utilization of a state immunization registry would allow ED providers to correctly identify a patient’s vaccination status. Reliably determining a patient’s vaccination status could potentially reduce unnecessary tetanus vaccine administration in patients with tetanus-prone wounds. Our objective was to determine whether use of an online immunization registry would impact the provision of tetanus prophylaxis for pediatric patients managed in the ED.

**Population Health Research Capsule**

What do we already know about this issue?

Emergency physicians must assess a patient’s need for tetanus prophylaxis, but often the patient’s vaccine status is unknown.

What was the research question?

Can an online immunization registry improve the accuracy of determining a patient’s tetanus vaccine status in the ED?

What was the major finding of the study?

25% of patients could have been spared the tetanus vaccine if the immunization registry had been used.

How does this improve population health?

If an immunization registry were implemented in the ED, the costs of redundant tetanus vaccines to both the patient and the system could be saved.

## METHODS

We designed this study to retrospectively review the EHR of pediatric patients who received tetanus vaccination in the ED and compare the data from Florida SHOTS on vaccination status to determine whether vaccination was indicated at the time of presentation to the ED. The institutional review boards at both facilities approved this retrospective review to be conducted.

Trained research assistants performed chart review and data entry. We used the REDCap (Research Electronic Data Capture) application to store data in order to retrospectively review the EHR of all pediatric patients who received a tetanus vaccine in the ED from January 1, 2011–May 31, 2015. This also included the patients for whom the tetanus vaccine was ordered but who received it after leaving the ED (on the floor or in the intensive care unit). This included all forms of tetanus vaccines given to pediatric patients under age 18, including DTaP, DT, Dtap/Hepatitis B/Polio (Pediarix), Td, Tdap, and tetanus toxoid. Children who did not receive a tetanus vaccine (including those for whom it may have been indicated) were excluded due to the method of data extraction (children who received the vaccine).

We conducted the study at two EDs in Florida, located about 70 miles from each other. Both EDs are located within academic institutions, and there are approximately 20,000–25,000 pediatric visits to each site annually. Variables collected for patients included age, gender, chief complaint, insurance status, primary care provider, and registration status in Florida SHOTS. The Florida SHOTS database was accessed to determine vaccination status at the time of the ED visit. This was compared to documentation about vaccination status in the hospitals’ EHRs. Of note, the EHR vaccination status was obtained from the immunization tab, which can be updated by physicians and/or nurses at any time, at any visit to the hospital or a clinic within the same system. Both nursing documentation and physician documentation about tetanus vaccination status were also examined by reviewing the notes in the EHR.

We performed descriptive statistics to summarize demographic variables. Documentation of the pediatric patients’ vaccination status in both the EHR and Florida SHOTS at the time of ED encounter were reported in frequencies and percentages. We performed all data analysis using SAS version 9.4 software (SAS Institute, Cary, NC).

## RESULTS

We identified 703 patients who received some form of tetanus prophylaxis in the ED. Of those patients, 438 (62.3%) were seen at the first site, and 265 (37.7%) were seen at the second site. Seventy percent of all patients were male, and the median age was 12.4 years old. Fifty-three percent were White, and 38% were Black; 58% were Medicaid patients, and 10% were self-pay. Sixty percent of the patients reportedly had a “delayed” vaccination schedule, according to the EHR. Most of the chief complaints fell into the category of laceration/wound/puncture (73%), and the remaining complaints were burns, trauma, and other. The EHR documented that 487 patients (70%) had a primary care provider (PCP), 175 (25%) did not, and 37 (5%) were unknown. This relates to our data because if a PCP is reported, immunization data is more likely to be documented in the online vaccination registry. The primary care physician’s office is typically responsible for updating the vaccination registry. In this group of 703 patients, only 2.5% (18 children) were not registered in Florida SHOTS. The demographics are summarized below in Table 1.

When discussing the results, “up to date” indicates that the child’s tetanus vaccination status was current, and the tetanus vaccine was not indicated during the ED visit; thus, the vaccine was unnecessarily administered. “Not up to date” means that the tetanus vaccine was indicated and thus, appropriately administered. As stated previously, all the children in the study received the tetanus vaccine in the ED (except for 15 of the patients, or about 2%, who were given the vaccine subsequently during the hospitalization after it was ordered in the ED).

Interestingly, we collected data from both the nursing notes as well as the physician notes in the EHR. Nursing documentation reported 481 (69%) patients were “up to date,” 90 (12%) were “not up to date,” and 129 (18%) were “unknown.” Physician documentation reflected 85 (12%) as “up to date,” with 383 (54%) as “not up to date,” and 234 (33%) as “unknown.” The breakdown by site was similar to the overall results. The reasons for these differences are unclear, but it highlights the issue of discrepancies in obtaining the vaccination status of patients in the ED and the need for a vaccination registry with more accurate information.

We examined whether the Florida SHOTS data (patient’s entire vaccine record) appeared in the EHR at the time of the ED visit. It was not present in 386 (56%) of the records, but 303 (44%) did contain the complete Florida SHOTS data in the EHR. There was a large discrepancy between sites: Site 1’s EHR contained the Florida SHOTS data only 25% of the time, while Site 2’s EHR contained the Florida SHOTS data 75% of the time. We also reviewed whether the tetanus vaccine given in the ED was documented in the Florida SHOTS record: 281 (41%) of the Florida SHOTS records did not contain documentation of the tetanus vaccine given in the ED, and 410 (59%) did contain the vaccine administered in the ED. Again, there was wide variability here with Florida SHOTS containing documentation of the tetanus vaccine given at Site 1 only 50% of the time, whereas it documented those given at Site 2 73% of the time.

The EHR review reflected that 43 (6%) of patient records were listed as “up to date” and 656 (93%) patient records were listed as “not up to date,” thus requiring a tetanus vaccine. When comparing Florida SHOTS data, 209 (30%) patients were listed as “up to date” (not requiring vaccine), and 477 (70%) were “not up to date” (did require vaccination). Of the 209 patients who were listed as “up to date” in Florida SHOTS, only 35 of them were documented as being “up to date” in the EHR as well. This means that 174 (25% of the entire patient population) patients were documented as “up to date” in Florida SHOTS but as “not up to date” in the EHR. These patients likely received the tetanus vaccine unnecessarily. This data is shown by site in Table 2, and the summary data is outlined in Table 3 below. It is important to note that patients for whom the tetanus vaccination status was missing from the EHR and/or Florida SHOTS were marked as “not up to date.”

## DISCUSSION

As mentioned above, in this group of 703 patients, only 2.5% of the patients (18 children) were not registered in Florida SHOTS. These patients may not have been Florida residents. The other 97.5% of the children registered in Florida SHOTS had the potential to benefit from the vaccination registry. About 70% of the children were noted to have a PCP. This is significant because the PCP’s office is the primary site where data is documented into Florida SHOTS. There was a slight discrepancy between the sites: 75% of the patients at Site 1 had a PCP, while only 60% at Site 2 had a PCP.

As mentioned above, EHR documentation showed that 43 (6%) patient records were listed as “up to date” and 656 (93%) patient records were listed as “not up to date,” thus requiring a tetanus vaccine. It is unclear why the patients in the group of 43 (6%) were administered a tetanus vaccine when the EHR indicated that they were already up to date. One reason this may have occurred is that the risk of the injury may have been so great that an additional vaccine was administered due to the high concern for developing tetanus. Additionally, it is possible that the tetanus vaccine was given during the initial trauma resuscitation, prior to family members arriving to provide the vaccine history.

According to Florida SHOTS records, the tetanus vaccine was indicated and administered appropriately to the majority of the pediatric patients who received the tetanus vaccine in our ED settings (70%). However, almost a third of the patients studied may have received the vaccine unnecessarily. Additionally, there were several discrepancies between the EHR and Florida SHOTS records. There were even larger discrepancies between nursing and physician documentation within the EHR. For patients who received the vaccine unnecessarily, there were likely multiple factors that led to the vaccine unnecessarily being administered. This may include the non-utilization of Florida SHOTS at the time of administration. Florida SHOTS does require a login, and while the nurses in the primary care clinics routinely access this resource, the ED nurses may not have access or be appropriately trained to access Florida SHOTS. Additionally, the ED is inherently busy, so time was likely a factor for both nurses and physicians deciding to access Florida SHOTS. In the academic hospital settings for this study, there are also multiple residents from different backgrounds (pediatrics, emergency medicine [EM], family medicine), and not all of them have access to Florida SHOTS, which would also have contributed to their inability to verify immunization status. Pediatric residents who also work in the pediatric continuity clinics have access to Florida SHOTS. However, the EM and family medicine residents did not have access.

It is also important to note that 40% of these patients who received the tetanus vaccine in the ED never had their Florida SHOTS records updated to reflect this. The ED providers do not routinely update Florida SHOTS with immunizations provided in the ED. It is therefore up to the PCP’s office to complete this task. However, many patients do not have a PCP or may not follow up with them after an ED visit. If they do follow up, they may forget to report that they received the vaccine, especially if it is not noted in their discharge paperwork. This is important because these patients may then receive the vaccine in the PCP’s office when the booster was previously scheduled to be due, or if they sustain another injury, they may again receive the vaccine in the ED unnecessarily.

Over 30% of patients receiving the tetanus vaccine in error translates to major costs for both the patients and the hospitals. The pediatric population is especially sensitive to painful injections and often requires extra measures, such as involving child life specialists to make the experience less traumatic. They may require an extra person to help hold them while the injection is being administered. Besides requiring the extra attention from busy ED personnel, each medication administered comes with a monetary cost. Compared to outpatient costs for medications and vaccines, costs in the ED are substantially higher. These costs may not be covered by insurance, and they can add to the family’s financial burden.

Since the completion of this study, the EHR integrated Florida SHOTS directly into its immunizations section so that the Florida SHOTS records are automatically updated in the EHR when accessed. This will likely decrease the discrepancies between the EHR and Florida SHOTS and possibly decrease rates of inappropriate administration of the tetanus vaccine. This change was made in 2017; so it would be interesting to examine the data after another 1–2 years.

Interestingly, a prospective adult study in Rome comparing patients’ memory to a rapid immunochromatographic test (Tetanus Quick Stick [Nephrotek Lab, Rungis, France) found that the TQS was able to save unnecessary tetanus vaccines 57% of the time.^6^ A similar study of 200 adults showed that almost 40% of them had incorrect recall of their tetanus vaccination status.^7^ However, one contrasting adult study in France did find that patients self-reported that their tetanus vaccines were up to date correctly about 96% of the time.^8^ It may be interesting to pursue a prospective study in the pediatric ED comparing patients’/parents’ memories, EHR, and state vaccination registry to a tetanus rapid immunochromatographic test.

## LIMITATIONS

A limitation of this study was that only the medical records of patients who received the tetanus vaccine during their ED visit/hospitalization were reviewed. Therefore, we did not examine cases of children in which the tetanus vaccine may have been indicated but was not provided. This was a result of the selection of cases from the EHR by those for whom the tetanus vaccine had been ordered in the ED. Another limitation of the study was its retrospective design. A few patients were missing some of the data points because they were not recorded in the EHR (three patients were missing nursing documentation of tetanus status, and one was missing physician documentation). However, this is unlikely to have significantly affected the results. Also, it was not possible to determine with certainty why the discrepancies existed between the EHR and Florida SHOTS or even between the various medical personnel (nurses, physicians) taking care of the patient.

## CONCLUSION

This retrospective review of the electronic health records and the state vaccination registry of 703 pediatric patients seen at two EDs between 2011–2015 showed that 25–30% of them received tetanus prophylaxis when it was not indicated. Access to Florida SHOTS provides valuable information regarding vaccination status that impacts patient care and resource utilization in the ED. If the physicians and/or nurses were readily able to access the vaccination registry from the ED, the costs of the tetanus vaccine to the patient and system could be saved. In 2017 (after the conclusion of this study), Florida SHOTS was incorporated directly into the hospital’s EHR. This will likely decrease the number of patients receiving the tetanus vaccine unnecessarily. It would be interesting to review the data again after this change was implemented.

## Figures and Tables

**Table 1:** Demographic characteristics of pediatric patients receiving the tetanus vaccine in the emergency department.

Variables	Site 1 (n = 438)	Site 2 (n = 265)	Total (n = 703)
Age (years), median (IQR)	11.70 (8.60)	14.40 (6.35)	12.4 (6.9)
Gender, Number (%)			
Male	299 (68.26)	191 (72.08)	490 (69.7)
Female	139 (31.74)	74 (27.92)	213 (30.3)
Race, Number (%)			
White	288 (66.06)	83 (31.32)	371 (52.92)
Black	104 (23.85)	161 (60.75)	265 (37.8)
Other (Hispanic, Asian, Native American, Multiracial)	44 (10.09)	21 (7.93)	65 (9.28)
Payer Status, Number (%)			
Medicaid	226 (51.6)	185 (69.81)	411 (58.46)
Commercial	161 (36.76)	44 (16.6)	205 (29.16)
Self-Pay/Charity	41 (9.36)	26 (9.81)	67 (9.53)
Other	10 (2.28)	10 (3.77)	20 (2.84)
Chief Complaint, Number (%)			
Burn	25 (5.72)	6 (2.26)	31 (4.42)
Laceration, Wound, Puncture	305 (69.79)	205 (77.36)	510 (72.65)
Trauma Alert (Activated Level 1 or Level 2)	55 (12.59)	22 (8.3)	77 (10.97)
Other	52 (11.9)	32 (12.08)	84 (11.97)
Does patient have a primary care provider? Number (%)			
Yes	327 (75.35)	160 (60.38)	487 (69.67)
No	105 (24.19)	70 (26.42)	175 (25.04
Unknown	2 (0.46)	35 (13.21)	37 (5.29)

*IQR*, interquartile range.

**Table 2:** Comparison of tetanus vaccination status in the electronic health record and Florida SHOTS for the pediatric patients receiving the tetanus vaccine by site.

	Site 1 (n = 438)	Site 2 (n = 265)	Total (n = 703)
According to EHR tetanus dates, did the patient need a vaccination? Frequency (Percentage)			
No (Up to date)	39 (8.99)	4 (1.51)	43 (6.15)
Yes (Not up to date)	395 (91.01)	261 (98.49)	656 (93.85)
According to Florida SHOTS tetanus dates, did the patient need a vaccination? Frequency (Percentage)			
No (Up to date)	159 (36.81)	50 (19.69)	209 (30.47)
Yes (Not up to date)	273 (63.19)	204 (80.31)	477 (69.53)
Immunization status - per nursing documentation Frequency (Percentage)			
Up to date	289 (66.44)	192 (72.45)	481 (68.71)
Not up to date	75 (17.24)	15 (5.66)	90 (12.86)
Unknown	71 (16.32)	58 (21.89)	129 (18.43)
Immunization status (per physician documentation)			
Up to date	72 (16.48)	13 (4.91)	85(12.11)
Not up to date	171 (39.13)	212 (80)	383(54.56)
Unknown	194 (44.39)	40 (15.09)	234(33.33)
Was tetanus vaccination given in the ED? Frequency (Percentage)			
No	15 (3.43)	0	15 (2.14)
Yes	422 (96.57)	265 (100)	687 (97.86)
Tetanus vaccine type given in ED, Frequency (Percentage)			
DTaP	85 (19.77)	18 (6.79)	103 (14.82)
DT	4 (0.93)	6 (2.26)	10 (1.44)
DTap, Hepatitis B, Polio (Pediarix)	2 (0.47)	10 (3.77)	12 (1.73)
Td	16 (3.72)	40 (15.09)	56 (8.06)
Tdap	286 (66.51)	191 (72.08)	477 (68.63)
Tetanus Toxoid (Booster)	37 (8.6)	0	386 (56.02)
Does the Florida SHOTS vaccination data appear on the EHR Immunizations record? Frequency (Percentage)			
No	319 (75.06)	67 (25.38)	386 (56.02)
Yes	106 (24.94)	197 (74.62)	303 (43.98)
Does the vaccination from the date of ED encounter appear on Florida SHOTS? Frequency (Percentage)			
No	211 (49.53)	70 (26.42)	281 (40.67)
Yes	215 (50.47)	195 (73.58)	410 (59.33)

*EHR*, electronic health record; *Florida SHOTS*, Florida State Health Online Tracking System; *ED*, emergency department; *D*, diphtheria; *T*, tetanus; *aP*, acellular pertussis. The case indicates amount of each ingredient in the vaccine.

**Table 3:** Overall comparison of tetanus vaccination status in the electronic health record and Florida SHOTS for the pediatric patients receiving the tetanus vaccine.

	According to FL SHOTS tetanus dates, did the patient need a vaccination? Frequency (Percentage)			P-value
According to EMR Tetanus Dates, did the patient need a vaccination? Frequency (Percentage)	No/Up to date	Yes/Not up to date	Total	<0.001
No/Up to date	35 (5.10)	8 (1.17)	43 (6.27)	
Yes/Not up to date	174 (25.36)	469 (68.37)	643 (93.73)	
Total	209 (30.46)	477 (69.54)	686 (100)	

Note: p-value was calculated using chi-square test.

*EHR*, electronic health record; *SHOTS*, State Health Online Tracking System.
